# Exploring optoelectronic properties and mechanisms of layered ferroelectric K_4_Nb_6_O_17_ nanocrystalline films and nanolaminas

**DOI:** 10.1038/s41598-017-01838-6

**Published:** 2017-05-15

**Authors:** Qinglin Deng, Mengjiao Li, Junyong Wang, Peng Zhang, Kai Jiang, Jinzhong Zhang, Zhigao Hu, Junhao Chu

**Affiliations:** 1 Technical Center for Multifunctional Magneto-Optical Spectroscopy (ECNU), Shanghai, China; 2 Department of Electronic Engineering, East China Normal University, Shanghai, 200241 China

## Abstract

Two-dimensional layered K_4_Nb_6_O_17_ (KN) was easily formed as a secondary phase caused by the volatilization of alkali metal ions, when preparing ferroelectric K_*x*_Na_1−*x*_NbO_3_ based ceramics and films. In this work, it was believed that KN film is with weak ferroelectricity and has a little effect on the ferroelectric properties of K_*x*_Na_1−*x*_NbO_3_ based films. Moreover, temperature dependent (77–500 K) dielectric functions of KN film have been firstly extracted by fitting ellipsometric spectra with the Adachi dielectric function model and a four-phase layered model. The high-frequency dielectric constant linearly increases and optical band gap slightly decreases with increasing the temperature. We also research its photoelectrochemical properties and its application in high-efficient light-induced H_2_ evolution. In addition, X-ray photoelectron spectroscopy, Raman scattering, temperature dependent transmittance and infrared reflectance spectra, and first-principles calculation were conjointly performed to further reveal the intrinsic optoelectronic features and relevant mechanisms of KN.

## Introduction

Since the discovery of graphene, two-dimensional (2D) layered materials such as metal chalcogenides, black phosphorus, transition metal oxides, and other 2D materials have raised great research interest due to their unique optical and electrical properties^1–5^. Among them, transition metal oxides possess numerous huge advantages, which can be prepared under oxygen atmosphere and endure high temperature^6, 7^. As a typical case, layered K_4_Nb_6_O_17_ (abbreviated as KN) has attracted considerable concern owing to its charming features and widespread applications in energy conversion, environmental purification, catalysis, non-linear optics, etc.^8–11^. KN is composed of octahedral units of NbO_6_ which forms a 2D layered structure via bridging oxygen atoms. There alternately are two different types of interlayer regions, denoted by interlayer I and interlayer II, which have different properties on hydration and ion exchange^12–17^.

As an effective and typical photocatalytic material^6, 18–20^, KN has been the subject of extensive studies on its photochemical and ion exchange properties^9, 21–25^. On the one hand, for global energy crisis, hydrogen is widely recognized as the fuel of the future, and photocatalytic water splitting into hydrogen has received significant attention^26–30^. Therefore, high-efficient and multifunctional materials for H_2_ production are urgently desired. KN can be synthesized by various methods, especially the conventional solid-state reaction method, which was frequently adopted to prepared KN for the studies of photocatalytic hydrogen production^19, 31, 32^. However, other methods such as hydrothermal method is relatively less to be reported. Considering that there are a few reports on modified hydrothermal method focusing on the photocatalytic H_2_ production for KN, so it raise that the hydrogen production rate are urgently desired to improve^9^. Moreover, these relevant kinetics mechanisms should be detailedly investigated to further guide the photocatalytic performances of KN-based family and other similar layered semiconductor materials.

On the other hand, the research of KN film, which can be used to build miniaturized devices, is relatively less. It was noteworthy that, KN was easily formed as a secondary phase caused by the volatilization of alkali metal ions, when preparing the K_0.5_Na_0.5_NbO_3_ film in our previous study^33^. Moreover, this phenomenon also frequently occurs in the preparation of ferroelectric KNbO_3_ and K_*x*_Na_1−*x*_NbO_3_ based ceramic and film^34–36^. KN has an orthorhombic structure with space group *P*2_1_ *nb*, and its single crystal was reported to show weak ferroelectricity^13, 37–39^. However, it is ambiguous about the ferroelectricity of KN film. In addition, it was believed that KN would bring some interferences during measuring the ferroelectric and optical properties in KNbO_3_ or K_*x*_Na_1−*x*_NbO_3_ based materials. Thus, the optoelectronic properties of its films become a key research issue need to be solved urgently, such as its ferroelectricity, infrared and ellipsometric spectra. Note that spectroscopic ellipsometry (SE) is a powerful and nondestructive tool that allows us to simultaneously obtain the thickness and optical parameters of a multilayer system without the Kramers-Krönig transformation (KKT)^33, 40, 41^.

In this work, we devote much efforts to the preparation of KN nanocrystalline films deposited on different substrates, as well as KN nano powders, and investigate their relevant optoelectronic properties. Remarkably, as far as we know, both the ferroelectric properties and optical properties studied by SE of KN film were investigated at the first time. Moreover, we also research its photoelectrochemical properties and its application in high-efficient light-induced H_2_ evolution. In addition, the studies of X-ray photoelectron spectroscopy (XPS), Raman scattering, infrared (IR) reflectance spectra, and first-principles calculation were also performed. It is believed that the present work could be helpful in deeply understanding the intrinsic features of KN and developing potential multifunctional KN-based applications.

## Results and Discussion

### Crystal structure

**Figure 1:**
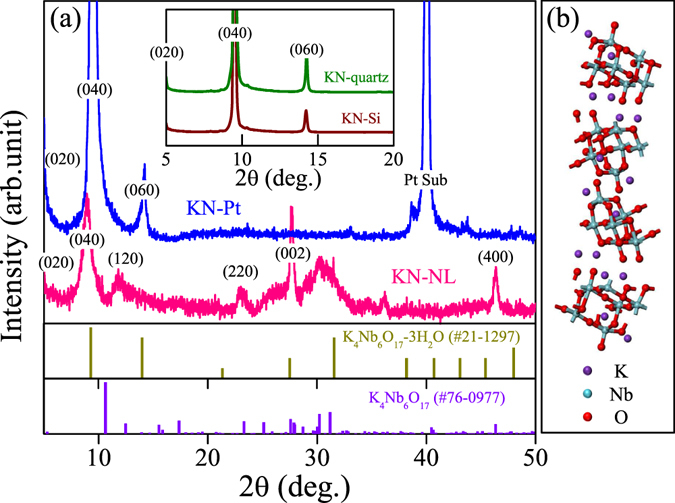
(**a**) XRD patterns of KN-NL, KN-Pt, KN-Si and KN-quartz films. (**b**) Simulated structures of KN.

The X-ray diffraction (XRD) patterns of powder and film samples are shown in Fig. 1a. After carefully checking the diffraction peaks of KN-NL and KN-Pt with different standard powder XRD patterns, it was believed that the XRD patterns of KN-NL and KN-Pt were more like K_4_Nb_6_O_17_ (JCPDS No. 76-0977) and K_4_Nb_6_O_17_-3H_2_0 (JCPDS No. 21-1297), respectively. However, both KN-NL and KN-Pt were hydrated, which results in the obvious shift of some diffraction peaks. The (040) diffraction peak of KN-Pt was located on a higher 2*θ* than KN-NL, which indicated that the content of water molecule of KN-Pt was less than KN-NL. Note that KN has interlayer I and interlayer II, and KN-*x*H_2_O is easily formed by adsorbing moisture in air^13^. Typically, the (040) diffraction peaks are always slightly different with the standard card. The *d*_040_ spacing is the interlayer spacing of KN. So it can be understood that the (040) diffraction peaks of hydrothermal derived KN-NL and sol-gel derived KN-Pt show a shift. Moreover, as compared with KN-NL, the diffraction peaks of KN-Pt were discrepant, for example, it did not show the peaks near 2theta 28 and 32 degree. Generally, the film samples are usually preferred orientation along the substrate to grow. For KN-Pt, it indicates that the preferable effect of the KN particles with the *b*-axis perpendicular to the substrate. This phenomenon can be found in other reports^13^. For the very strong peak at 40 degree, it can be attributed to the peak of Pt substrate. The weak and broadening diffraction peaks for KN powder samples suggest that the obtained products might be composed of small crystal grains with the size in nanoscale. The inset of Fig. 1a shows the XRD patterns of KN-Si and KN-quartz films. Obviously, both of them display a strong and sharp characteristic (040) reflection, which is in good agreement with other studies^13^. Figure 1b shows the supercell of KN orthorhombic structure. As can be seen, the unique layered structure of KN is composed of (Nb_6_O_17_)^4−^ layers stacked along the *b* axis and K^+^ ions occupy two different interlayer regions^38^.

### Surface morphologies and ferroelectric properties

**Figure 2:**
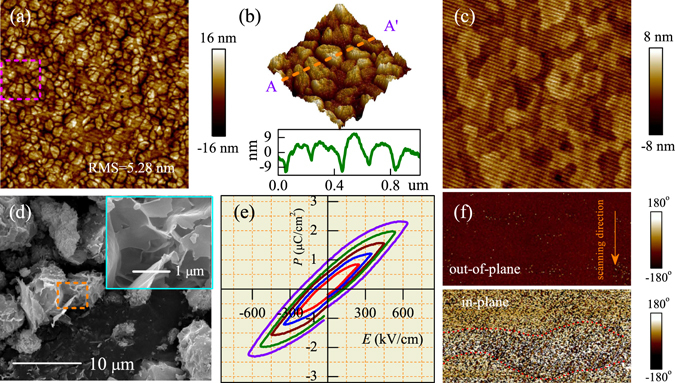
(**a**) 2D AFM images of KN-Pt, note that the measured area is 5 × 5 *μ*m^2^. (**b**) 3D AFM image of KN-Pt is the enlargement of the dashed box in (**a**), and the measured area is 1 × 1 *μ*m^2^. Note that the inset data shows the section analysis of the 3D AFM image based on the AA′ area. (**c**) 2D AFM images of KN-quartz, note that the measured area is 1 × 1 *μ*m^2^. (**d**) SEM images of KN-NL. (**e**) Polarization-electric field (*P*-*E*) ferroelectric hysteresis loops of KN-Pt. (**f**) The phase of IPP and OPP signals for the KN-Pt film. Note that the short dash areas indicate the possible domain, and the measured area is 500 × 250 nm^2^.

Figure 2a shows the 2D AFM image of KN-Pt film. The root-mean-square (RMS) surface roughness is about 5.28 nm. The three-dimensional (3D) AFM image in Fig. 2b shows the enlargement of the dashed box in Fig. 2a. It can be seen that the substrates are covered with nanoparticles, which indicates that the film has nanocrystalline growth patterns. Note that the inset data in Fig. 2b shows the section analysis of the 3D AFM image based on the AA′ area. It was found that the KN-Pt film has the average grain size of about 200 nm. The surface morphology of the KN-quartz film has been also investigated by AFM (shown in Fig. 2c). The RMS surface roughness of the KN-quartz film is about 2.0 nm, and it also has nanocrystalline growth patterns. Due to the lattice matching effects of different substrate, KN-quartz film has a more smooth surface than the KN-Pt film. In addition, the morphologies of the powder samples were characterized by scanning electron microscopy (SEM). As shown in Fig. 2d, the synthesized KN-NL exhibited aggregated lamina morphology from different magnifications.

In order to investigate the ambiguous ferroelectric properties of the KN film. The typical polarization-electric field (*P*-*E*) ferroelectric hysteresis loops of the Pt/KN/Pt capacitors were measured at *f* = 10 kHz and room temperature. The relevant data are shown in Fig. 2e. It can be found that the KN-Pt film has weak ferroelectricity, which is in good agreement with the report on KN single crystal^39^. As an example, a relatively smaller 2 *P*_*r*_ of 1.9 *μ*C/cm^2^ was obtained in the KN-Pt film at an applied electric field of 630 kV/cm. Note that the Mn doped K_0.5_Na_0.5_NbO_3_ (KNN) film deposited on Pt substrate shows a larger 2 *P*_*r*_ of 31 *μ*C/cm^2^ at an applied electric field of 600 kV/cm in our previous study^33^. For KN single crystal, Kimura *et al*. found that a relatively smaller 2 *P*_*r*_ of about 1.1 *μ*C/cm^2^ was obtained at an applied electric field of 2 kV^39^. The value of 2 *P*_*r*_ for KN-Pt film is slightly larger than KN single crystal. The reason can be attributed to the different applied electric field. Moreover, due to the dielectric loss derived from the hydrated KN-Pt film, thus the actual 2 *P*_*r*_ of KN-Pt was lower than 1.9 *μ*C/cm^2^. In addition, in our work the piezoresponse force microscopy (PFM) experiments have been carried out to investigate the polar nano-domain behavior of KN-Pt. As shown in Fig. 2f, by combining the study of in-plane polarization (IPP) and out-of-plane polarization (OPP) images, a not very clear domain contrast can be observed in the in-plane direction. The domain contrast is not obvious, the reason might be attributed to the very weak ferroelectricity of KN-Pt.

Indeed, KN-Pt prepared in the present work contains lattice defects, which can result in the dielectric loss. Only the data including *P*-*E* loops and PFM analysis, cannot fully guarantee the ferroelectricity of KN. However, combining with the similar research results of KN single crystal by H. Kimura *et al*.^39^, it was believed that KN has weak ferroelectricity. Therefore, it was believed that the KN-Pt film is of weak ferroelectricity and has a little effect on the ferroelectric properties of KNN-based films.

### XPS analysis

**Figure 3:**
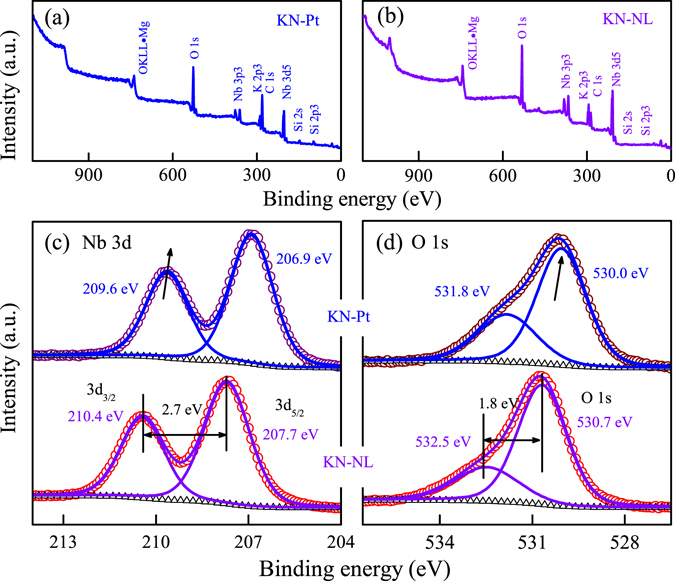
The typical overall XPS spectrum of (**a**) KN-Pt and (**b**) KN-NL. High-resolution XPS spectra of (**c**) Nb 3d and (**d**) O 1 s for KN-Pt and KN-NL.

The chemical state of the contained elements for KN samples were investigated by using XPS technique. As an example, Fig. 3a,b present the typical overall XPS spectrum of KN-Pt and KN-NL, respectively. As we can see, K, Nb and O elements can be detected on the surface. As shown in Fig. 3c, it was found that the Nb 3d XPS spectra of KN-NL were observed at 210.4 eV and 207.7 eV for the 3d_3/2_ and 3d_5/2_ peaks, respectively, and the distance between the two peaks is 2.7 eV. This unquestionably suggests that the Nb ion is in the 5+ valence state, which is in good agreement with the findings in the core level spectra of Nb_2_O_5_^42^. The binding energy of 3d_3/2_ and 3d_5/2_ peaks for KN-Pt shift toward to lower energy. It might be attributed to the effects derived from morphology and substrate. Note that the distance between these two peaks of Nb 3d for KN-Pt remains the same of about 2.7 eV. Figure 3d shows the high-resolution XPS spectra of O 1 s. It was found that the O 1 s peaks for KN-NL with binding energies located at 532.5 eV and 530.7 eV, were assigned to the hydroxyl oxygen and lattice oxygen, respectively. And the distance between these two peaks is 1.8 eV. Moreover, the binding energy of the two peaks for KN-Pt shift toward to lower energy, which is similar to the phenomenon of Nb 3d.

### Lattice vibrations

**Figure 4:**
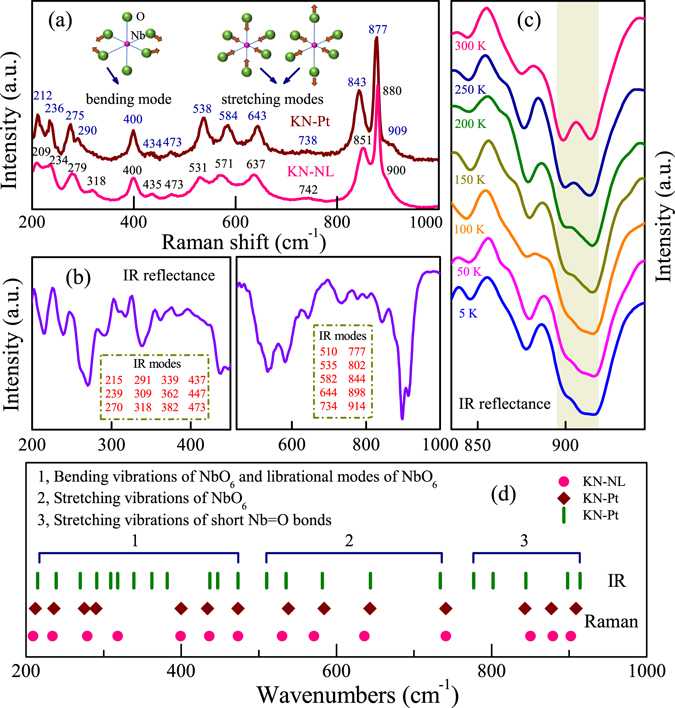
(**a**) Raman spectra of KN-Pt and KN-NL. The insets show the schematic illustrations of bending and stretching vibrational modes of NbO_6_ octahedra. (**b**) IR reflectance spectra of KN-Pt at room temperature. (**c**) Temperature dependent IR reflectance spectra of KN-Pt. The rectangular area corresponds to the obvious changes of IR modes. (**d**) Comparison of Raman and IR active modes for KN-NL and KN-Pt with the proposed assignment.

Raman scattering is sensitive to the change in the coordination of local symmetry, which gives information about the molecular vibrations and distortions of the crystal lattice^33, 40, 43^. IR reflectance spectroscopy is an attractive and powerful tool for optical characterization of semiconductor materials^44, 45^. Thus, the combined use of both two techniques could further investigate the structural features of KN. Theoretically, KN has an orthorhombic structure with space group *P*2_1_ *nb*. The unit cell contains four formulas that have 324 zone-centre degrees of freedom described by the irreducible representation 81*A*_1_ + 81*A*_2_ + 81*B*_1_ + 81*B*_2_^46^. The NbO_6_ octahedra mainly gives rise to the stretching and bending vibrations modes. As shown in Fig. 4a, it can be seen that both KN-Pt and KN-NL show sharp and strong Raman bands around 877 cm^−1^, which is in good agreement with other studies^47^. For KN-Pt, the Raman bands at 843, 877, 909 cm^−1^ and 538, 584, 643 cm^−1^ were assigned to the stretching modes of the shorter and longer niobium oxygen bonds, respectively. The remaining modes in the 200–480 cm^−1^ range correspond to bending vibrations of the NbO_6_ octahedra. As we can see, there are some obvious changes in the Raman bands of KN powder and film. For instance, we do not observe the obvious split peaks at the characteristic frequency of about 280 cm^−1^ for KN-NL, however, KN-Pt show two peaks around this wavenumber. As we all know, due to the weak interactions in the direction perpendicular to the layers, the NbO_6_ octahedra are easily distorted, which may lead to the obvious changes of some Raman bands. Detailedly, for KN-NL, some Raman bands have significant broadening as compared with KN-Pt, for example, the bands at 209, 234, 531, 571 and 631 cm^−1^. The reason may be attributed to the intercalation of more water molecules. The Raman bands at 843, 877, 909 cm^−1^ for KN-Pt, exhibit about −8, −3 and +9 cm^−1^ shifts as compared with KN-NL. Even more pronounced shifts are also observed for the Raman bands of KN-Pt at 738, 643, 584 and 538 cm^−1^ (−4, +6, +13 and +7 cm^−1^, respectively, as compared with KN-NL). In the lattice and bending modes region, significant shifts are also observed for the Raman bands in the 200–350 cm^−1^ range. The observed shifts of the Raman bands indicate that hydrated effects lead to significant changes of some Nb-O bonds and angles. Note that XRD results reveals that both KN-NL and KN-Pt were hydrated, the (040) diffraction peak of KN-Pt was located on a higher 2*θ* than KN-NL, which indicated that the content of water molecule of KN-Pt was less than KN-NL. Taking the preferable effect of KN-Pt into consideration, it was believed that KN-Pt was more like KN single crystal film. Due to the expansion of the interlayer distances derived from the intercalation of more water molecules, it leads to some bands of KN-NL shift towards lower wavenumbers. The research results described in our work are in good agreement with the report by M. Maczka *et al*.^46^.

Figure 4b displays the IR reflectance spectra of KN-Pt. The main reflection bands are marked in the IR spectra, which is in accordance with other studies^46, 48^. For comparison, Raman and IR active modes with the proposed assignments are plotted in Fig. 4d. To further reveal the lattice vibrations of KN, the temperature dependent IR reflectance spectra were recorded in the range of 5–300 K, as shown in Fig. 4c. It demonstrates that the changes in intensity and peak position of the corresponding bands at 914 and 898 cm^−1^ are very sensitive to temperature variation. The reason is interpreted as follows, due to weak interactions in the direction perpendicular to the layers, the NbO_6_ octahedral units might be strongly distorted by the variable temperature, resulting in the obvious changes in intensity and peak position of some short Nb-O bonds.

**Figure 5:**
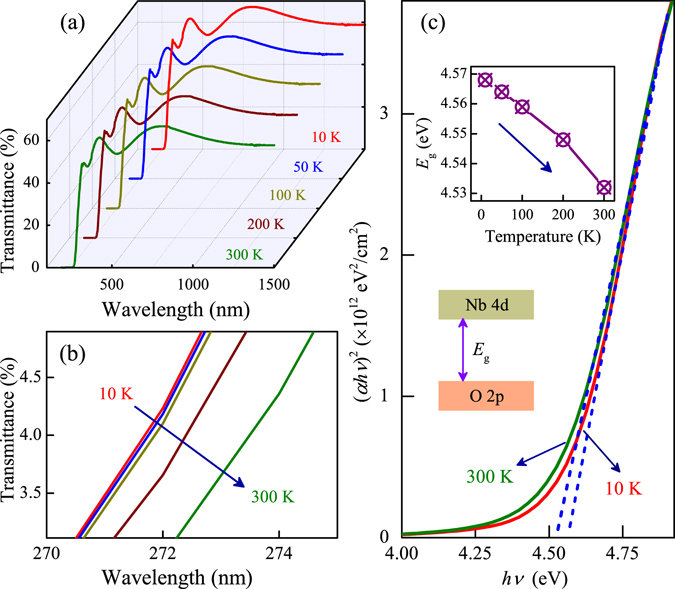
(**a**) Temperature dependent transmittance spectra of KN-quartz film. (**b**) An enlarged spectral region near the absorption edge for KN-quartz film at different temperatures. (**c**) Plots of (α*h*ν)^2^ ν*s*. the photon energy for the estimation of direct optical band gap energies for KN-quartz film at 10 and 300 K. Note that the inset in (**c**) is the temperature dependent *E*_g_ of KN-quartz film and the schematic representation of the electronic band structure of KN.

In addition, we also study the temperature dependent transmittance spectra of the KN-quartz film, as shown in Fig. 5a. It can be clearly seen that each film has a relatively high optical transmittance in the visible range. The enlarged transmittance spectra from 10 K to 300 K for the KN-quartz film near the absorption edge are shown in Fig. 5b. Obviously, the absorption edge shows a redshift trend with increasing the temperature. In order to understand the temperature dependent band-gap behavior in detail, the optical band gap energies were investigated. As we all know, it can determine the absorption band gap energy using the acknowledged Tauc formula^49–51^, expressed by (α*h*ν)^*n*^ = K(*h*ν − *E*_g_), where α is the absorption coefficient, *h*ν is the photon energy, K is a constant relative to the material and n depends on whether the transition is direct (n = 2) or indirect (n = 1/2). The optical transition for KN is directly allowed (seen in the section of theoretical study), so the value of n for KN is 2. Fig. 5c shows the values of the direct optical band gaps for KN-quartz film at different temperature. Obviously, the values slightly decrease with increasing the temperature, which is based on the well-known temperature effects.

### Ellipsometric spectra

**Figure 6:**
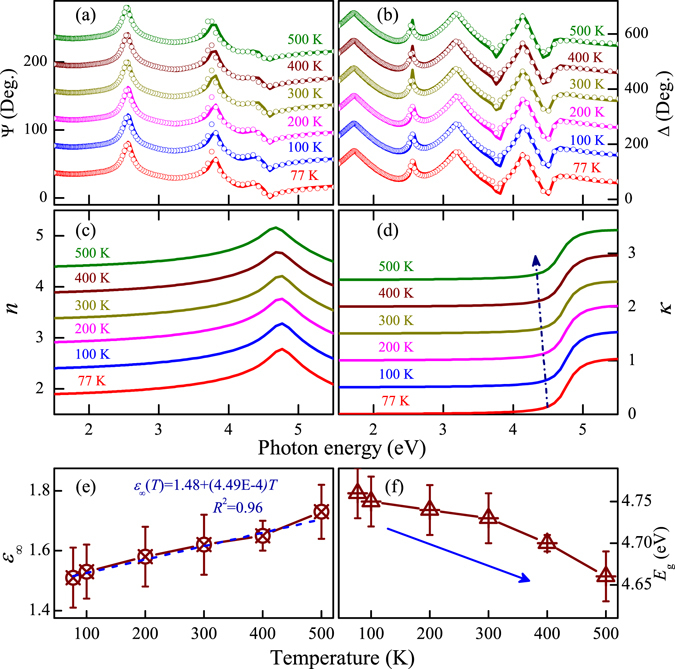
The NIR-UV experimental (dotted lines) and best-fitted (solid lines) ellipsometric spectra (**a**) Ψ(*E*) and (**b**) Δ(*E*) of KN-Pt under 77, 100, 200, 300, 400 and 500 K. Evolution of (**c**) refractive index (*n*) and (**d**) extinction coefficient (κ) of KN-Pt with increasing the temperature. For clarity, the arrow indicates the absorption edge redshift with increasing the temperature. Note that Ψ, Δ, *n* and κ are vertically shifted by adding 40, 100, 0.5 and 0.5, respectively.

Generally, based on the reflectance configuration, spectroscopic ellipsometry (SE) provides a sensitive and non-destructive method to extract simultaneously optical constants and thicknesses of multilayer systems^33, 41^. In order to further investigate the optical dispersion behavior of KN, temperature dependent dielectric functions of KN-Pt have been investigated by SE from 77 K to 500 K. Note that SE measures relative changes in the amplitude Ψ(*E*) and phase Δ(*E*) of particular directions polarized lights upon oblique reflection from the sample surface. With regard to wide band gap semiconductor materials, the complex dielectric functions $\tilde{{\rm{\varepsilon }}}$(*E*) can be expressed by the Adachi model, which is based on the KKT and connected with the electronic band structure. The method reveals the distinct structures at energy of the M_1_-type critical point, which is written as: ${\rm{\varepsilon }}(E)={{\rm{\varepsilon }}}_{\infty }-{A}_{0}ln(1-{{\rm{\chi }}}_{0}^{2})/{{\rm{\chi }}}_{0}^{2}$, ${{\rm{\chi }}}_{0}=(E+i{{\rm{\Gamma }}}_{0})/{E}_{{\rm{g}}}$. Here, ${{\rm{\varepsilon }}}_{\infty }$ is the high-frequency dielectric constant, *A*_0_ and ${{\rm{\Gamma }}}_{0}$ are the strength and broadening values of the optical band gap (*E*_g_) transition, respectively. The thickness of film (*d*_f_) and SRL (*d*_s_), *E*_g_ and ${{\rm{\varepsilon }}}_{\infty }$ could be extracted from the best fit between the experimental and theoretical spectra. Correspondingly, the optical constants [$\tilde{N}(E)=n(E)+i{\rm{\kappa }}(E)$, refractive index (*n*) and extinction coefficient (κ)] can be calculated from the well-known relationships $\tilde{N}(E)$ = $\sqrt{\tilde{{\rm{\varepsilon }}}(E)}$. As shown in Fig. 6a,b, a good agreement is obtained between the experimental and fitted ellipsometric spectra. It confirms that the selected Adachi dielectric function model (CPM1)^52^ and the four-phase layered structure (air/surface rough layer (SRL)/KN-Pt/Pt substrate) can be reasonable and acceptable for KN-Pt. Moreover, the fitted model parameter values are summarized in Table 1. In the transparent region: *E* < *E*_g_ (optical band gap), the κ (Fig. 6d) is closer to zero and then remarkably increases as the photon energy further increases beyond the fundamental band gap. It suggests that a strong optical absorption appears, which shows the interband electronic transition from the top of the valence band to the conduction band for KN-Pt.

**Table 1:** The parameter values of the Adachi dielectric function model for KN-Pt determined from the simulation of ellipsometric spectra in Fig. 6a,b.

Temperature	ε_∞_	*A*_0_	Γ_0_ (eV)	*E*_g_ (eV)	*d*_s_ (nm)	*d*_f_ (nm)
77 K	1.51 ± 0.10	1.98 ± 0.09	0.127 ± 0.012	4.76 ± 0.03	3.1 ± 1.3	193.2 ± 4.3
100 K	1.53 ± 0.09	1.99 ± 0.07	0.129 ± 0.009	4.75 ± 0.03	2.6 ± 1.1	192.3 ± 4.6
200 K	1.58 ± 0.10	1.97 ± 0.05	0.134 ± 0.009	4.74 ± 0.03	1.7 ± 0.9	191.3 ± 4.2
300 K	1.62 ± 0.10	1.83 ± 0.05	0.125 ± 0.011	4.73 ± 0.03	2.1 ± 1.3	197.4 ± 4.2
400 K	1.65 ± 0.05	1.84 ± 0.07	0.140 ± 0.010	4.70 ± 0.01	1.3 ± 1.2	197.2 ± 3.0
500 K	1.73 ± 0.09	1.78 ± 0.07	0.141 ± 0.009	4.66 ± 0.03	0.8 ± 1.2	196.6 ± 3.1

Note that the 95% reliability of the fitting parameters is given with (±).

The Adachi model parameters ${{\rm{\varepsilon }}}_{\infty }$ and *E*_g_ with the fitted values are shown in Fig. 6e,f, respectively. It can be seen that ${{\rm{\varepsilon }}}_{\infty }$ linearly increases with increasing the temperature. The *E*_g_ slightly decreases with increasing the temperature, which is in accordance with the results of transmittance spectra in KN-quartz film. The average *E*_g_ of the KN-Pt is 4.7 eV. It should be emphasized that the value is much larger than that derived by the theoretical calculation (2.95 eV, in this work) and experimental results (3.61 eV for KN-NL in our work). It can be ascribed to the huge stress and imperfect crystallinity in the thin KN-Pt film. The similar enlarged band gap was also observed in other ferroelectric oxides^33, 53^. From the viewpoint of KN-based capacitor devices and ultraviolet detectors, a wider optical band gap material can be suitable for these potential applications.

### Photocurrent and photocatalytic activities

**Figure 7:**
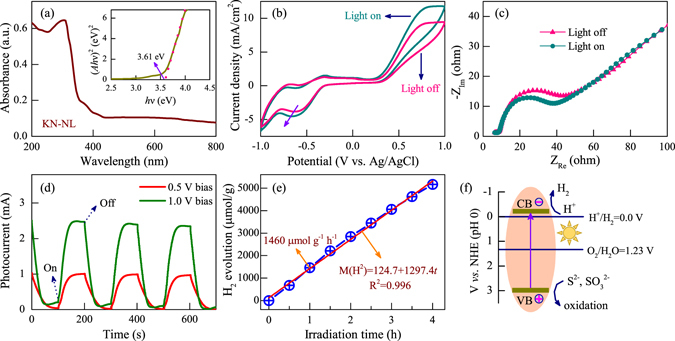
(**a**) UV-VIS DRS of KN-NL with the inset curve of (*Ah*ν)^2^ ν*s*. *h*ν. (**b**) CV curves and (**c**) EIS Nyquist plots of KN-NL in an aqueous solution based three-electrode test under light irradiation and dark. (**d**) Photocurrent responses of KN-NL electrodes under chopped illumination with different bias voltage. (**e**) The amount of H_2_ evolved from as-prepared KN-NL photocatalyst under different irradiation time. (**f**) The proposed mechanism for photocatalytic H_2_ production.

As we know, the band gap structure of the semiconductor plays a key role in photocatalytic activity. UV-VIS diffuse reflectance spectra of KN-NL is illustrated in Fig. 7a. The spectra clearly show the characteristic absorption edge. The band gap (*E*_g_) of the KN-NL is estimated to be 3.61 eV, from (*Ah*ν)^2^ ν*s*. *h*ν plots (inset in Fig. 7a). Generally, the cyclic voltammetry (CV) and electrochemical impedance spectra (EIS) techniques based three-electrode test have been used to investigate the charge transfer process^54^. As shown in Fig. 7b, it can be found that KN-NL shows the enhanced current density under light irradiation as compared with that under dark, which indicates the improved electron transfer rate and separation efficiency of photoinduced carriers. In addition, KN-NL show semicircles at high frequencies according to EIS Nyquist plots (shown in Fig. 7c). The arc radius of the EIS Nyquist plot of KN-NL under light irradiation is smaller than that under dark, suggesting that KN-NL may has a decrease in charge-transfer resistance under light irradiation. The CV and EIS results indicate that KN-NL could obtain a relatively high photocurrent response value. In order to further investigate the carrier separation abilities of KN-NL, photocurrent responses measurements were carried out under chopped illumination with different bias voltage. As shown in Fig. 7d, it was found that the photocurrent responses are reversible as the illumination is turned on and off. With increasing the bias voltage, the photocurrent value increases accordingly. The photocurrent response is not fast, the reasons might be attributed to the large electrode coating area and defective electrode contact. However, in our work, it demonstrates that the photocurrent can reach a larger value of about 2.36 mA under applying a 1.0 V bias.

The above research results have attested that KN-NL would performed good photocatalytic activity, as the excellent electron transfer rate and separation efficiency of photoinduced carriers. It helps us to screening out more efficient catalyst for splitting water into hydrogen. In this work, photocatalytic hydrogen evolution of KN-NL was evaluated under UV-VIS light irradiation using aqueous solution containing Na_2_S and Na_2_SO_3_ as a scavenger. As shown in Fig. 7e, KN-NL exhibits high-efficient photocatalytic H_2_ production activity in absence of a cocatalyst. The amount of H_2_ evolution increases with increasing the irradiation time, and it can be well expressed by the linear relationship: 124.7 + 1297.4 *t*. It has a high H_2_ production rate of about 1460 *μ*mol h^−1^ g^−1^. The apparent quantum yield (AQY) can be estimated as AQY(%) = 200 R/I, where R and I represent the amount of H_2_ evolved and number of photons impinge on the sample, respectively. Figure 7f shows the proposed mechanism for photocatalytic H_2_ production. Firstly, the electrons originating from KN-NL jump from the valence band (VB) to the conduction band (CB) under light irradiation. Then the hydrogen ions accept the electron to produce hydrogen, while the holes in VB can be consumed by the sacrificial agents (S^2−^ and ${{\rm{SO}}}_{3}^{2-}$). Note that most of the reports on photocatalytic hydrogen production of KN are focused on using conventional solid-state reaction method to prepare KN^18, 19^. We use the modified hydrothermal method to prepare KN-NL, then investigate its photocatalytic activity. As some typical examples, K. Domen *et al*. have prepared KN by solid state reaction method, and obtain a high H_2_ production rate of about 250 *μ*mol h^−1^ g^−1^ ^23^. K. Maeda *et al*. also used the solid state reaction method to prepare 0.1 wt% Pt-loaded KN, and a high H_2_ production rate of about 720 *μ*mol h^−1^ g^−1^ was obtained^19^. Moreover, C. Zhou *et al*. have prepared self-doped KN microspheres by a hydrothermal method. They obtained a high H_2_ production rate of about 1100 *μ*mol h^−1^ g^−1^ under UV light irradiation^9^. As we can see, KN-NL prepared in our work has a relatively higher H_2_ production rate of about 1460 *μ*mol h^−1^ g^−1^, which indicates that KN-NL shows good photocatalytic activity. The main reason might be attributed to the unique porous structure, with faster mass transport and more accessible active sites, resulting in an increased photocatalytic reaction rate. Although some noble-metal based catalysts show a higher H_2_ production activity, the use of noble metal is high-cost^55–57^. In order to achieve a wide range of application, catalysts made from rich and low cost materials are urgently desired. On the one hand, KN-NL is composed of earth-abundant Nb and K elements. On the other hand, KN-NL was prepared by a mild and simple hydrothermal process. Therefore, it can be considered as one of the most promising candidates for the high-efficient light-induced H_2_ production by water splitting.

### Theoretical study

It has been verified that using first-principles calculation on ferroelectric materials not only can further explore the essential features but also guide the experiments for potential applications^58–60^. In the present work, the plane-wave-based density-functional theory (DFT) calculation with the generalized gradient approximation (GGA) was carried out for pure KN (orthorhombic phase^38^) to obtain the information about the energy structure. The Perdew-Burke-Ernzerhof (PBE) functions are used to address exchange-correlation interactions along with a standard plane-wave basis set with a kinetic-energy cutoff of 400 eV. These calculations are performed by using the 3 × 1 × 4 Monkhorst-Pack *k*-point mesh, and the convergence criterion for the electronic energy is 10^−5^ eV.

**Figure 8:**
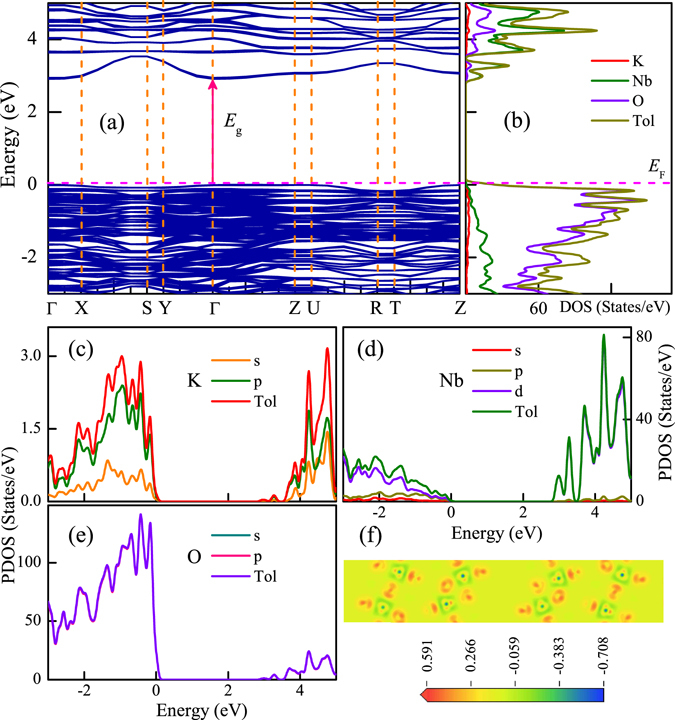
(**a**) Calculated band structure and (**b**) corresponding density of states for KN, where *E*_F_ denotes the Fermi energy level. Note that the solid arrow located at the point of symmetry ${\rm{\Gamma }}$ indicates the direct electron transitions of optical band gap. Partial density of states for (**c**) K, (**d**) Nb and (**e**) O elements, respectively. (**f**) Charge density difference of KN.

Figure 8a,b show the calculated electronic band structure and density of states (DOS) of pure orthorhombic KN from −3 eV to 5 eV. The highest occupied state was chosen as the Fermi level (*E*_F_) and set to zero as the reference. As we can see, the solid arrow located at the point of symmetry ${\rm{\Gamma }}$ in Fig. 8a indicates the direct electron transition of optical band gap, which is approximately 2.95 eV. As a well-known problem of calculations based on DFT, the calculated optical band gaps are underestimated by about 20–30%, as compared to the experimental values^40^. So it is understandable that the value is smaller than that derived by the experimental results (3.6 eV for KN-NL). According to the DOS of KN and the partial density of states (PDOS) of K, Nb and O elements, as shown in Fig. 8b–e, it is clear that the top of valence bands (VBs) and bottom of conduction bands (CBs) are composed of mainly O-2p and Nb-4d states, respectively. The s and p states of K have little contribution to the VBs and CBs. Moreover, combining the studies of photocatalysis, it can be concluded that the photo-induced electrons were excited from O-2p states to Nb-4d states. Note that the ${{\rm{\varepsilon }}}_{\infty }$ of KN linearly increases with increasing the temperature (depicted in the SE analysis), which indicates that the contributions from the high-energy interband transitions become more prominent with increasing the temperature. On the basis of DOS and PDOS results, these high-energy interband transitions might be ascribed to the transitions that from the states of O-2p to the hybridization states of Nb-4d with O-2p. In addition, as shown in Fig. 8f, the charge density difference of KN clearly depicts the strong interaction of Nb and O atoms due to the overlap of atomic orbitals.

## Conclusion

In conclusion, we have successfully synthesized KN nanocrystalline films on Pt, Si and quartz substrates, as well as KN nanolaminas. Due to the effects of morphology and substrate, KN films and powders show unique microstructures and optoelectronic properties. The Adachi dielectric function model and a four-phase layered model were successfully applied to fit the ellipsometric spectra and reasonably described the optical dispersion behavior of the KN-Pt film, which is with weak ferroelectricity (proved in this work). Moreover, KN-NL has been proved to be a promising photocatalyst for the high-efficient hydrogen production by water splitting. In addition, the corresponding spectrum studies and theoretical calculation further reveal the lattice vibrations and electronic structure of KN. Therefore, we conclude that our comprehensive work for KN not only can be helpful in exploring the optoelectronic properties of other similar layered oxides, but also provides a vital reference information for investigating KNbO_3_ and K_*x*_Na_1−*x*_NbO_3_ based films.

## Methods

### Preparation of KN powders

All of the chemical reagents and solvents used in the experiments were purchased from commercial sources and without any further purification. The KN nanolaminas (KN-NL) were prepared by a mild hydrothermal process. Typically, 2.4 g Nb_2_O_5_ powder was added into 80 mL 0.9 mol/L KOH solution. The resulting mixture was transferred into a 100 mL polytetrafluoroethylene-lined stainless autoclave and hydrothermally treated at 200 °C for 12 h, followed by natural cooling to room temperature. The products were obtained by centrifugation, washed several times, and finally dried in a vacuum oven at 80 °C for 24 h.

### Preparation of KN films

The experimental procedures can be described as follows. Raw materials of potassium acetate (CH_3_COOK) and niobium ethoxide [(CH_3_CH_2_O)_5_Nb] were mixed in stoichiometric composition. Acetic acid and 2-methoxyethanol (CH_3_OCH_2_CH_2_OH) were used as solvent. Acetylacetone (CH_3_COCH_2_COCH_3_) was used as chelating agent. To compensate the loss of alkaline metals during thermal annealing, 10 mol% K excess was added to precursor solution. The precursor solutions were magnetically stirred at a constant temperature bath. Then the sol-gel derived (K, Nb)-precursor (SG-precursor) were obtained by adjusted to 0.2 mol/L and filtered by using 0.22 *μ*m pore size filter. To obtain KN-Pt films, the SG-precursor solutions were spin-coated onto Pt(111)/Ti/SiO_2_/Si(100) substrate. Prior to the deposition, the substrates need to be cleaned in pure acetone and ethanol with an ultrasonic bath to remove oily substance and other impurities from the surfaces, followed by rinsing several times with DI water, then the substrates were dried in a pure nitrogen stream. The films were deposited by employing a spin-coating process at a speed of 4000 rpm for 20 s. Each layer of the films was dried at 200 °C for 200 s to evaporate the solvent and then pyrolyzed at 400 °C for 200 s to remove residual organic compounds, followed by annealing at 700 °C for 300 s to crystallize the films by a rapid thermal annealing procedure. The spin-coating and annealing-treatment procedures were repeated ten times to obtain a desired thickness. The KN-Si and KN-quartz films, which were deposited on Si and quartz substrates, respectively, can be obtained by following the above procedures.

### Characterization methods

The crystal phase structures of all samples were analyzed by X-ray diffraction (XRD, Cu Kα, D8 Advance, Bruker). X-ray photoelectron spectroscopy (XPS) measurements were carried out on a RBD upgraded PHI-5000C ESCA system (Perkin-Elmer) with Mg-Kα radiation (*h*ν = 1253.6 eV), and binding energies were calibrated by using the containment carbon (C 1 s = 284.6 eV). The surface morphologies of powder and film samples were examined by field emission scanning electron microscopy (FESEM: Philips XL30FEG) and atomic force microscopy (AFM: Digital Instruments Icon, Bruker), respectively. The domain behavior is based on the PFM modes of AFM. Raman scattering experiments were carried out using a micro-Raman spectrometer (Jobin-Yvon LabRAM HR 800UV). In order to get rid of the trivial temperature dependence, all Raman spectra have been divided by the Bose-Einstein occupation number n(ω) + 1 = 1/[1 − exp(−*ħ*ω/*k*_B_ *T*)] (*ħ* and *k*_B_ are Planck constant and Boltzmann constant, respectively). Ultraviolet-visible diffuse reflectance spectra (UV-VIS DRS) were recorded by a double beam infrared-ultraviolet spectrometer (Perkin-Elmer UV/VIS Lambda 950) equipped with an integrating sphere assembly. Platinum (Pt) dots with the diameter of about 0.2 mm deposited by sputtering technique using a shadow mask are used as top electrodes for electrical measurements, while the Pt layer was served as a bottom electrode. The hysteresis loops of the KN-Pt films were measured by a ferroelectric test system (Precision Premier II: Radiant Technologies, Inc.) at *f* = 10 kHz and room temperature. The temperature dependent infrared reflectance spectra of the KN-Pt films were recorded by a Bruker VERTEX 80 V Fourier transform infrared (FTIR) spectrometer. The transmittance spectra at 10–300 K were measured by using a double beam ultraviolet-infrared spectrophotometer (Perkin Elmer UV/Vis Lambda 950). The samples were mounted on a cold stage of an optical cryostat (Janis SHI-4-1).

The ellipsometric spectra measurements were carried out by a near-infrared to ultraviolet (NIR-UV) SE in the photon energy range of 1.5–5.5 eV with a spectral resolution of 5 nm (V-VASE by J. A. Woollam Co., Inc.). The incident angle was selected as 70° for the films. As for the variable temperature measurements, the samples were fixed into Janis CRV-275V with liquid nitrogen cooling accessory and the temperature can be set from 77 K to 500 K with a precision of about ±1 K. Note that the window effect can be eliminated through the calibration of silicon sample.

Photoelectrochemical tests were implemented on a CHI660E electrochemical workstation with a three-electrode system in an aqueous solution containing 0.5 mol/L Na_2_SO_4_ and some ascorbic acid. Cyclic voltammetry (CV) experiments were performed at a scan rate of 100 mV/s. The as-prepared KN-NL were used as a working electrode, a platinum sheet as the counter electrode and Ag/AgCl as the reference electrode. The working electrodes were prepared on FTO substrates by adding appropriate amount of a slurry, which consisted of 80 wt% active materials, 10 wt% acetylene black and 10 wt% polyvinylidene fluoride (PVDF) dissolved in N-methyl-2-pyrrolidinone (NMP), then dried in a vacuum oven at 90 °C for 24 h. Electrochemical impedance spectroscopy (EIS) were performed by using an impedance measurement unit of the electrochemical workstation in the frequency range of 0.1–10^5^ Hz, with an ac amplitude of 5 mV. Photocurrent measurements were carried out under chopped illumination from a 300 W Xe-lamp.

For photocatalytic H_2_ production experiments, 100 mg of KN-NL was dispersed in an aqueous solution (100 mL) containing 0.12 mol/L Na_2_S and 0.12 mol/L Na_2_SO_3_. The reaction solution was evacuated several times to remove H_2_ completely prior to irradiation. Then the solution was irradiated using a 300 W Xe-lamp without an optical filter. The temperature of the reaction solution was maintained at room temperature by a flow of cooling water. The evolved gases were sampled out every 30 minute. The gases carried by Ar were analyzed by gas chromatograph equipped with a thermal conductive detector (TCD) and a 5 *Å* molecular sieve column.
